# Clinical Impact of Next-generation Sequencing in Pediatric Neuro-Oncology Patients: A Single-institutional Experience

**DOI:** 10.7759/cureus.6281

**Published:** 2019-12-03

**Authors:** Valentin Barsan, Megan Paul, Hamza Gorsi, Denise Malicki, Jennifer Elster, Dennis J Kuo, John Crawford

**Affiliations:** 1 Pediatric Hematology/Oncology, Stanford University School of Medicine, Palo Alto, USA; 2 Pediatric Hematology/Oncology, University of California San Diego, La Jolla, USA; 3 Pediatric Hematology/Oncology, Children's Hospital of Michigan, Michigan, USA; 4 Pathology, University of California San Diego, La Jolla, USA; 5 Neurosciences and Pediatrics, University of California San Diego, La Jolla, USA

**Keywords:** next-generation sequencing, pediatric brain tumors, precision medicine, targeted therapy

## Abstract

The implementation of next-generation sequencing (NGS) in pediatric neuro-oncology may impact diagnosis, prognosis, therapeutic strategies, clinical trial enrollment, and germline risk. We retrospectively analyzed 58 neuro-oncology patients (31 boys, 27 girls, average age 7.4 years) who underwent NGS tumor profiling using a single commercially available platform on paraffin-embedded tissue obtained at diagnosis (20 low-grade gliomas, 12 high-grade gliomas, 11 embryonal tumors, four ependymal tumors, three meningeal tumors, and eight other CNS tumors) from May 2014 to December 2016. NGS results were analyzed for actionable mutations, variants of unknown significance and clinical impact. Seventy-four percent of patients (43 of 57) had actionable mutations; 26% had only variants of uncertain significance (VUS). NGS findings impacted treatment decisions in 55% of patients; 24% were given a targeted treatment based on NGS findings. Seven of eight patients with low-grade tumors treated with targeted therapy (everolimus, trametinib, or vemurafenib) experienced partial response or stable disease. All high-grade tumors had progressive disease on targeted therapy. Forty percent of patients had a revision or refinement of their diagnosis, and nine percent of patients were diagnosed with a previously unconfirmed cancer predisposition syndrome. Turnaround time between sample shipment and report generation averaged 13.4 ± 6.4 days. One sample failed due to insufficient DNA quantity. Our experience highlights the feasibility and clinical utility of NGS in the management of pediatric neuro-oncology patients. Future prospective clinical trials using NGS are needed to establish efficacy.

## Introduction

The genetic drivers of pediatric cancers are rapidly becoming better understood. Detection of genetic variants by next-generation sequencing (NGS) enables physicians to tailor therapeutic strategies for each patient. Incorporating genetic profiling can both enhance diagnostic accuracy and provide a mechanistic rationale for the selection of conventional and novel therapies. However, to date, the use of NGS in pediatric oncology clinical practice is not standardized, and its utility and limitations are not fully understood.

Identifying tumor genetic variants may increase specificity in pathologic diagnosis and lead to changes in patient management. In cancers of indistinct histology, variants can define subsets of tumors that have distinctive biological features and clinical characteristics which then inform prognosis [1]. This information qualifies further discussions regarding the treatment intensity, specific agents chosen, expected toxicity, and goals of care. Detection of variants that confirm a cancer predisposition syndrome may inform the need for ongoing cancer surveillance and testing of family members.

The clinical use of NGS data in pediatric oncology is in its infancy, and best practices of when and how to use NGS are developing. While many variants have known clinical implications, the prognostic significance of recurring genomic lesions for many cancers remains undefined. As compared to adult cancers, pediatric cancers exhibit “calmer” genomic landscapes with lower mutational frequencies and are distinctive from the molecular alterations observed in adult tumors [2]. Consequently, only a few variants implicated in pediatric tumors have an approved targeted therapy in any tumor type.

Many pediatric central nervous system (CNS) tumors, such as high-grade gliomas, have poor prognoses and few effective treatment options. These patients therefore potentially have much to gain from the incorporation of NGS into clinical practice. While NGS has advanced our understanding of pediatric CNS tumors, the gene content breadth, stage at clinical presentation, and actionability of genome-driven oncology care remain variable [3]. We, therefore, sought to perform a retrospective analysis of NGS utilization in pediatric neuro-oncology patient care at our institution over a two-year period using a single commercially available platform. We review how NGS has been utilized in our clinical cohort and discuss implications for utilization management and increasing clinical impact.

## Materials and methods

Fifty-eight non-consecutive patients with primary CNS tumors who had surgical resection or biopsy at the Rady Children’s Hospital, San Diego between May 2014 and December 2016 underwent NGS testing using a single commercially available platform. The selection of patients for NGS testing was by the treating neuro-oncologist. Selection for NGS was due to (1) the uncertainty of diagnosis by histology alone, (2) failure of established treatment options and screening for targetable mutations, and (3) atypical tumor behavior, such as an unexpected rate of progression of low-grade tumors. NGS profiling was performed by a Clinical Laboratory Improvement Amendments (CLIA)-certified laboratory (Foundation Medicine, Cambridge, MA) through either the FoundationOne® (N = 39) or FoundationOne Heme® (N = 19) panels. Specimens underwent a pathologic evaluation, and formalin-fixed paraffin-embedded (FFPE) sections were sent for NGS analysis. The laboratory and computational methods employed in the NGS assays have been previously described [4]. DNA extracted from FFPE samples underwent hybridization capture at predetermined genetic loci unique to each panel (FoundationOne® = 315 genes + 28 select rearrangements; FoundationOne Heme® = 405 genes + 31 select rearrangements + RNA sequencing of 265 genes). NGS at high depth (>500x) utilizing the Illumina HiSeq® for uniform sequencing coverage enabled the detection of all classes of genomic alterations including single-base substitutions, small insertions and deletions, rearrangements, and copy number alterations. The resulting report was reviewed by the treating physician and presented at neuro-oncology tumor board as part of routine clinical care. Actionable mutations were defined as those which altered diagnosis, altered treatment, or diagnosed a cancer predisposition syndrome. The timing of initiation of targeted therapy was by treating neuro-oncologist's discretion. The duration of follow-up was until patient death or through July 2019.

Through a retrospective study of the electronic medical record under institutional review board approval (University of California, San Diego), patients were assigned a unique study identification number linking the patient name and medical record number to clinical elements in the chart and molecular variants of the NGS report. Patient’s NGS results were then binned into categories of clinical actionability: 1. those affecting diagnosis, 2. those in whom a change was made in patient management and 3. those leading to a cancer predisposition syndrome diagnosis.

## Results

Our 58-patient cohort was composed of 31 females and 27 males with an average age of 7.4 ± 5.3 years at the time of surgical resection (range: four months to 19 years, median: 6.5 years). The NGS analysis included 20 low-grade gliomas, 12 high-grade gliomas, 11 embryonal tumors, four ependymal tumors, three meningeal tumors, and eight other CNS tumors (Table 1).

**Table 1:** Patient demographic and treatment characteristics NGS, next-generation sequencing; PD, progressive disease; SD, stable disease; PR, partial response; VUS, variants of uncertain significance

Gender	
Male	27
Female	31
Age at time of surgery	
Mean	7.4 years
Median	6.5 years
Range	4 months to 19 years
Time to next-generation sequencing (NGS) testing after surgery	
Median	1 year
Range	11 days to 11.5 years
Tumor type	
Low-grade glioma	20
High-grade glioma	12
Medulloblastoma	6
Atypical Teratoid Rhabdoid Tumor	3
Primaiive Neuroectodermal Tumor	2
Ependymoma	
Anaplastic	3
Myxo-papillary	1
Meningioma	3
Atypical central neurocytoma	2
Atypical schwannoma	2
others	4
Time to reporting (average)	13.4 days (SD±6.4)
Types of mutation (% total)	
Patients with any mutation	57 (100%)
Patients with actionable mutations	43 (74%)
Patients with actionable and variants of unknown significance (VUS)	42 (72%)
Patients with VUS only	15 (26%)
Mean number of actionable mutations	2.8 (1-23)
Mean number of VUS	10.7 (1-186)
Tumor Mutation Burden	
High	1
Low	56
Impact of NGS findings (% total)	
Targeted therapies used	14 (24%)
Change in radiation plan	18 (31%)
Refined diagnosis	23 (40%)
Diagnosed cancer predisposition syndrome	5 (9%)
Response to targeted therapy if used (% total therapies used)	
Four patients received two subsequent treatments	
PR	2 (11%)
SD	7 (39%)
PD	7 (39%)
Unable to evaluate	2 (11%)

The average time between the date of surgical resection or biopsy and the decision to pursue NGS was one year but varied widely (standard deviation 21 months, median: three months, mode: one month, range: 11 days to 11.5 years). This reflects both clinical heterogeneity and the ability to perform NGS on archived samples. The turnaround time between sample shipment and report generation averaged 13.4 days (standard deviation: 6.4 days). Only one sample failed due to insufficient DNA quantity in a patient with a low-grade brainstem glioma who underwent biopsy.

**Figure 1:**
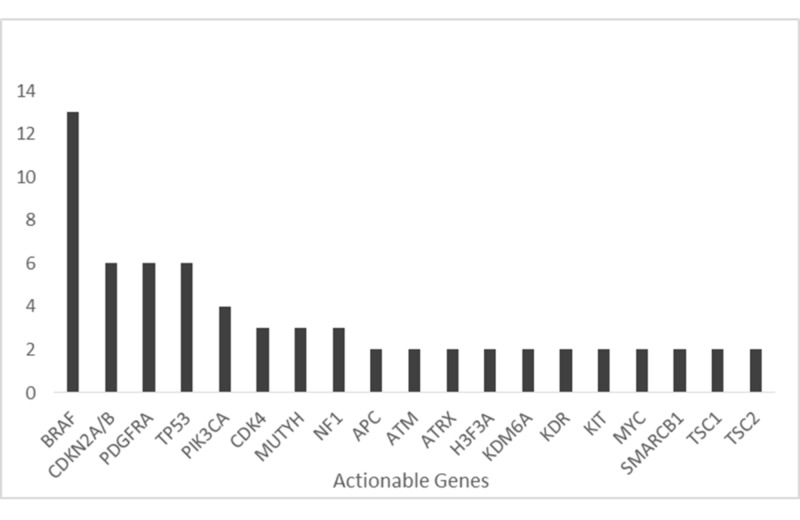
Most common actionable gene alterations detected and their frequencies The number of patients with each gene mutation is shown (*y*-axis). BRAF mutations are the most common actionable gene alterations detected. CDKN2A/B, PDGFRA, and TP53 mutations are all the second most common mutations. BRAF mutations include point mutations as well as fusion products.

Seventy-four percent (43/57) of samples that completed NGS testing were found to have “actionable” mutations as defined above, whereas the remaining 14 patients (26%) had only variants of uncertain significance (VUS) detected. Patients with actionable variants had an average of 2.8 actionable variants per report (standard deviation: 3.8, range: 1-23, mode: 1). Seventy-three genes were found to be actionable, 19 of which were detected at least twice (Figure 1).

Only 10 variants were detectable across more than one patient tumor in a consanguineous patient (Table 2).

**Table 2:** Most frequently observed genetic variants on next-generation sequencing (N = 57)

No	Gene	Variant	Count
1	BRAF	KIAA1549-BRAF fusion	7
2	BRAF	V600E	5
3	CDKN2A/B	loss	4
4	CDK4	amplification	3
5	MUTYH	G382D	2
6	SMARCB1	loss	2
7	KDR	amplification	2
8	KIT	amplification	2
9	PDGFRA	amplification	2
10	MYC	amplification	2

Four equivocal amplifications were detected by NGS in which the observed number of copies was on the verge of the FoundationOne® analytical threshold for copy number.

On average, 10.7 VUS per patient were detected by the NGS panels (range: 0-186). VUS were not utilized in clinical decision making, but some of these variants may have future clinical significance. Interestingly, one patient with 186 VUS had a presumptive history of neurofibromatosis type 1 (NF1) based on cutaneous stigmata but was found instead to have congenital mismatch repair deficiency (CMMRD) with an MSH6 mutation. This was the only patient with high tumor mutation burden (TMB) with 191 mutations per megabase. The remaining patients all had low TMB (seven or fewer mutations per megabase). Microsatellite instability was not detected in and of the 18 patients of which were analyzed.

The clinical impact of NGS sequencing included refining pathologic diagnosis, guidance in targeted agent choice, guiding use of radiation, and confirming a cancer predisposition syndrome (Table 1). NGS enabled a more refined diagnosis in 23 (40%) cases where pathologic workup was limited by unclear/mixed histology or quantity of tissue. Fourteen patients (24%) were given targeted therapy based on NGS results (Table 3).

**Table 3:** Next-generation sequencing findings, patient characteristics, and treatment responses of patients receiving targeted therapies post genetic analysis (N = 14) PD, progressive disease; SD, stable disease; PR, partial response

No	Patient history and histologic diagnosis	Mutation	Targeted therapy	Outcome
1	17 yo M low grade glioma	GLI1 P535S	Everolimus Trametinib	SD SD
2	6 yo F low grade glioma	BRAF-KIAA1549 fusion	Everolimus Trametinib	PD PD
3	6 yo F low grade glioma	BRAF-KIAA1549 fusion	Everolimus	SD
4	9 yo M low grade glioma	BRAF-KIAA1549 fusion	Everolimus Trametinib	SD PR
5	6 yo F low grade glioma	BRAF V600E	Everolimus	SD
6	8 yo F low grade glioma	EGFR R222C	Trametinib	PR
7	4 yo M Pleomorphic xanthoastrocytoma	BRAF V600E, TSC1 V831fs*22, CDKN2A/B loss	Vemurafenib Everolimus	SD
8	10 yo M pilocytic astrocytoma and NF1	NF1 splice site 2251+1G>A and splice site 3708+1G>A PDGFRA A491T	Everolimus and Trametinib	SD
9	12 yo F embryonal tumor with molecular features of glioma	AKT3 amplification BRAF-KIAA1549 ATM R457* IKBKE amplification IRS2 amplification MCL1 amplification FGF14 amplification GABRA6 T113M MDM4 amplification NOTCH2 P6fs*27 PIK3C2B amplification	Everolimus, Trametinib	PD
10	9 yo M high grade glioma	KDR amplification KIT amplification MET amplification PDGFRA amplification CDKN2A/B loss TP53 rearrangement intron 1	Pazopanib, Everolimus	PD
11	13 yo M atypical teratoid rhabdoid tumor	SMARCB1 loss CD36 Y325*	Alisertib	PD
12	9 yo F high-grade glioma	DNMT3A R635W FGFR3 E627D PDGFRA S1042L PIK3CA R88Q ATM splice site 8151+1G>A NOTCH1 V1599M TP53 R175* and R273C TYK2 R243W APC R348* CDH1 splice site 687+2T>C ELP2 R636Q ERBB2 R677* FOXP1 R525* HIST1H2AM T77fs*24 LRP1B C881* MEF2C R189* MLL2 R5021* MSH6 R841fs*3 NCOR2 R1590* RAD50 R656* SETD2 R2024* TMEM30A Q42* ZMYM3 splice site 711+2T>C	Pazopanib	PD
13	3 yo M high-grade glioma	CCND2 amplification CDK4 amplification MDM2 amplification	Palbociclib Trametinib	PD Unable to evaluate, not tolerated
14	5 yo M high-grade glioma	PIK3CA E545K	Everolimus	PD

**Figure 2:**
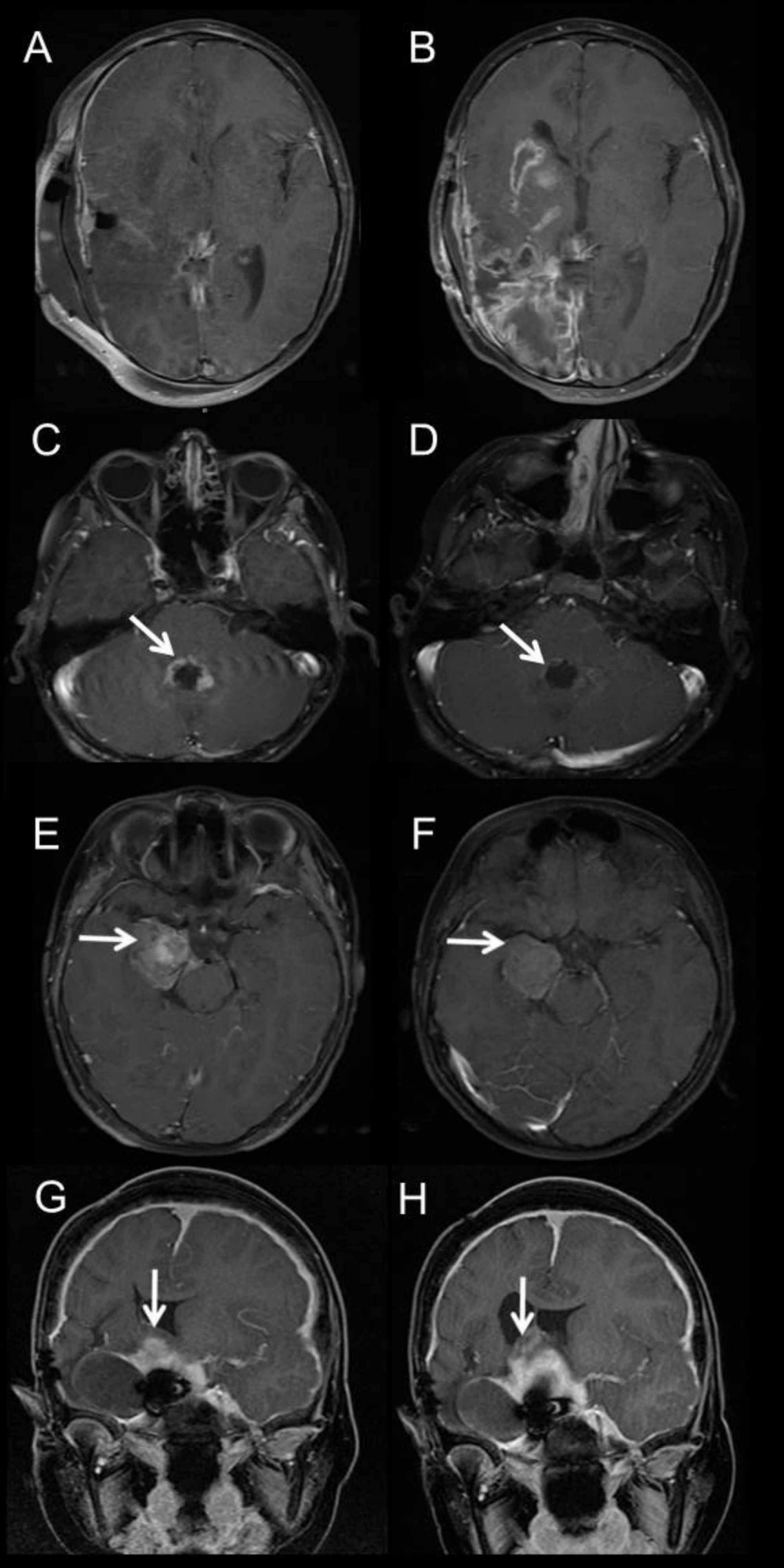
Magnetic resonance imaging findings pre- and post-targeted therapy A: Pre-targeted treatment (palbociclib and trametinib) MRI on a three-year-old boy with high-grade glioma (amplification of CCND2, CDK4, and MDM2 amplification). B: Post-treatment MRI showing progressive disease one month after therapy. C: Pre-treatment (everolimus and trametinib) MRI in a 10-year-old male with NF1 and multiply recurrent PDGFRA-mutated juvenile pilocytic astrocytoma of the posterior fossa (arrow). D: Post-treatment MRI reveals a near-complete response of the fourth ventricular tumor (arrow). E: Pre-treatment (vemurafenib) MRI in a four-year-old boy with V600E-mutated pleomorphic xanthoastrocytoma of the right temporal lobe (arrow). F: Post-treatment MRI reveals partial response (arrow). G: Pre-treatment (trametinib) MRI in a 17-year-old male with a GLI 1, P53-mutant multiply recurrent suprasellar low-grade glioma (arrow). H: Post-treatment MRI reveals stable suprasellar disease (arrow). MRI, magnetic resonance imaging

Eighty-eight (7/8) percent of patients with low-grade gliomas who received targeted therapy had either a partial response or stabilization of their disease (Figure 2).

All patients with low-grade gliomas who received a targeted therapy had previously been treated with a standard-of-care chemotherapy regimen such as carboplatin and vincristine. The most commonly used drugs used were everolimus and trametinib either alone or in combination. Detectable mutations by NGS in patients with low-grade gliomas included V600E (*N* = 2), BRAF KIAA-1549 (*N* = 3) EGFR (*N *= 1), PDGFR (*N *= 1) and Gli 1 (*N *= 1). Patients receiving a targeted agent for high-grade tumors all experienced progressive disease. Radiation therapy was avoided in 18 cases (32%), where there was a lack of malignant molecular features based on NGS. Tumor NGS demonstrated mutations that were consistent with a previously suspected but unconfirmed cancer predisposition syndrome in four cases (NF1, neurofibromatosis type 2, Von Hippel Lindau syndrome, and Gorlin syndrome), confirmed CMMRD in one patient previously thought to have NF1 and helped exclude Carney complex, tuberous sclerosis complex, and NF1 in three cases.

## Discussion

Our experience shows a significant impact of molecular profiling on diagnosis, prognosis, and treatment and validates its feasibility within clinically meaningful timeframes. In another published single institutional experience, NGS similarly helped refine diagnosis, and 61% of patients in that cohort were found to have potentially targetable variants [5]. The clinical utility of broader sequencing beyond limited panels to exomes has been previously reported in pediatric low-grade gliomas [6]. Supported by our findings, we favor the use of broader panels and caution against only testing for variants with a known therapeutic intervention.

NGS clarified the diagnosis in 23 (40%) cases and was especially useful when histology was not definitive or tissue was limited. Molecular characteristics were often used to refine glioma classification, thereby improving prediction of patient outcomes and informing the selection of radiotherapy for more aggressive tumor types [7]. For example, more than 80% of pleomorphic xanthoastrocytomas and 30% to 50% of gangliogliomas have BRAF V600E mutations [8]. Approximately half of high-grade diffuse gliomas show epidermal growth factor receptor (EGFR) 7p12 amplification, and a subset of them (approximately 20%) have an intragenic deletion of exons 2-7 (EGFRvIII variant), which leads to a constitutively activated truncated receptor [9]. These associations increasingly classify histologic diagnoses by molecular features.

Molecular information provided more information for difficult-to-classify tumors, as has also been shown in a previous study [10]. Additionally, five patients had mutations detected in the tumor suggesting a cancer predisposition syndrome (Von Hippel Lindau syndrome, Gorlin syndrome, NF1 and NF2, and CMMRD), which were then confirmed in the germline. Most of these were previously suspected but not confirmed. This information then allows for any necessary genetic counseling or additional monitoring to be carried out. A limitation of this study is that there was only gene-specific germline sequencing done in those patients with suspicion of a well-characterized cancer predisposition syndrome. There may be unrecognized novel cancer predisposition mutations that are therefore not confirmed in the germline, as was seen in a prior study [5].

Fourteen percent of patients in our series were given targeted therapy based on their actionable variant profiles. The majority of these were in either low-grade gliomas that had progressive disease despite the standard of care or high-grade gliomas where no standard of care exists beyond radiation. Interpretation of the responses to targeted therapy in this cohort is limited to broader application, as there was significant heterogeneity between patients regarding the length of treatment and prior treatments received. Patients were non-randomly selected for NGS, so the responses to therapy in this group may not be applicable to a more inclusive patient cohort.

We expect that the number of variants which are actionable will grow considerably over time. For example, the now well-known driver genetic alterations for pilocytic astrocytoma and pilomyxoid astrocytoma have been characterized in the MAPK pathway (most commonly BRAF-KIAA1459 fusion, but also NF1 mutations, BRAF V600E mutation, BRAF intragenic deletions, and BRAF fusions with other partners). Aggregation of such molecular data sets has enabled novel insights into the pathophysiology of gliomas and has led to the recent introduction of new drugs, which target the MAPK pathway, either indirectly through MEK inhibition or directly with BRAF V600E-specific drugs such as vemurafenib [11]. Patients with low-grade gliomas have excellent long-term survival but may go through multiple treatment regimens for refractory disease. Therefore, in this group of patients, particularly, there is a long window of time for new treatments to be developed and subsequently used, even years after a patient first undergoes sequencing. More comprehensive sequencing at the time of initial molecular profiling may provide genetic information that can direct targeted therapy years later, thus avoiding the need for repeated biopsies. While the more common BRAF V600E and BRAF-KIAA1459 fusion mutations can be detected through non-sequencing technologies such as fluorescence in situ hybridization (FISH) or immunohistochemistry, these techniques will not detect the less common mutations observed in low-grade gliomas such as EGFR mutations or less common BRAF mutations. Therefore, it may be more efficient and cost-effective in low-grade glioma patients to pursue NGS more readily, particularly when considering the use of a targeted treatment agent.

Biomarkers of immunogenicity such as microsatellite instability and TMB require broader molecular panels to identify those patients most likely to respond to immunotherapies such as checkpoint blockade [12]. Due to the timeframe of this patient cohort, no patients in our group received a checkpoint blockade agent; however, this treatment strategy has become more widely used and better understood in the intervening years.

Effective use of clinical genomics is anticipated to require new and improved tools to ascribe pathogenic significance and therapeutic actionability across interventional prospective clinical trials. Various groups such as the INdividualized Therapy FOr Relapsed Malignancies in Childhood (INFORM) registry, the Individualized Cancer Therapy (iCat) Study, the Baylor Advancing Sequencing into Childhood Cancer Care (BASIC3) project, St. Jude’s Children’s Research Hospital (SJCRH) Genomes for Kids, Precision in Pediatric Sequencing (PIPseq), and the Children’s Oncology Group MATCH trial are each advancing precision medicine for children with cancer to increase knowledge about the genomics of diagnosis, treatment, and relapse [13-18].

Though a powerful tool, there remain limitations to our reported experience and the overall clinical use of NGS in neuro-oncology. Variability across methods limits direct comparison due to various sample preparation methods and libraries, rapidly evolving instrumentation, differences in approach (hot-spot versus whole gene versus whole exome versus cell-free DNA), different methods of analysis (coverage depth, read regions), informatic pipelines, manual curation of variant calls, comparison to germline reads, and final reporting methods. The breadth of genomic content surveyed and the depth at which variants are detected have a significant impact on limits of detection, cost, and interpretative effort. The definition of variant “actionability” varies across physicians, institutions, and payers. Reporting guidelines have been proposed to bin variants according to levels of clinical usefulness [19]. Nevertheless, the all-comers approach to molecular profiling of brain tumors in a single-institution cohort through retrospective review offers an opportunity to reflect on the circumstances in which NGS has demonstrated clinical utility.

While many patients had actionable variants detected, the frequency of variants differed by histology. For example, none of the four ependymomas had actionable variants detected. The quiet mutational landscape of many pediatric tumors suggests that more comprehensive (i.e. exome, genome, methylation) sequencing approaches will be required to identify relevant tumor variants. Methylation profiling has begun to supplement genomics in sub-classifying pediatric CNS tumors and is likely to become a useful element of the diagnostic evaluation of pediatric neuro-oncology patients, particularly in those in whom NGS does not provide actionable information [20]. The depth of sequencing required for establishing the limit of detection for any given variant may be dependent on tumor histology. For example, heterogeneity within the tumor is particularly relevant to high-grade gliomas although less so to other CNS tumors, such as medulloblastoma [21]. Shallow sequencing protocols may not detect subclonal tumor cell populations. Spatial and temporal clonal heterogeneity in pediatric CNS tumors and the relative contributions of detected molecular variants (as qualified by their corresponding variant allele frequencies) to treatment failure remains relatively unexplored. In addition, the brain tumor microenvironment has been described as a critical regulator of tumorigenesis and our use of NGS was not powered towards discerning microenvironment characteristics such as differential immune infiltrates that have been previously reported through RNA sequencing deconvolution methods [22]. While actionable mutations were identified in a substantial proportion of tumors, it remains possible if not likely that emergent tumorigenic properties exist at the metabolic, immunologic, epigenomic, and metagenomic levels. For example, malignant pediatric brain tumors commonly display alterations of epigenetic regulation [23]. Additional distinct hallmark properties in cancer may not be optimally co-measured clinically with NGS.

Our cohort consisted of only primary tumors. The genomic landscapes of tumors at relapse remain underexplored. For several of our cases, the variants detected through NGS at diagnosis offered therapeutic contingency plans upon relapse. The sequencing of a contemporary tumor sample at relapse is preferable yet not always clinically indicated. Often a second biopsy or resection is felt to have excess risk or minimal benefits and is deferred. However, there may be a case made for an additional surgical procedure if the treatment plan would be altered based upon novel NGS findings. Variants in the relapsed tumor may arise through tumor evolution that could indicate a new targetable lesion or resistance to specific treatment options. The time elapsed between tumor resection and molecular profiling may impact the clinical relevance of detected variants proportionate to tumor grade, with high-grade tumors evolving more rapidly. Blood-based assays (liquid biopsies) have been developed in other tumor types to address this limitation as well as to trend levels of circulating tumor DNA (ctDNA) for signs of molecular response or relapse [24]. Two critical areas for future discovery are defining the genomic landscape at relapse or progression, and optimizing the blood and CSF-based biomarkers for assessment such as minimal residual disease at the ctDNA level.

There is rapidly expanding interest in using sequencing data to optimize pediatric neuro-oncology patient care; however, at this time, its utilization is not standardized and only a small number of institutional series have described its use [25-30]. Panel NGS-based tumor profiling in our single-institution pediatric neuro-oncology patient cohort yielded a clinically significant finding in most cases. However, only a small subset received a matched therapy as a result, and clinically relevant data were obtained in a larger proportion of the cohort. NGS enabled comprehensive identification of targetable mutations, nuanced histopathologic diagnosis, informed prognosis, suggested constitutional and familial testing for cancer predisposition syndromes, and suggested molecular targets worthy of further study. The clinical impact of NGS in neuro-oncology patients portends a hopeful future of true precision medicine, in which diagnosis is definitive, ineffective or inappropriate therapies are avoided, and mechanistic treatment plans prolong durable responses. Ongoing clinical trials aim to clarify when and how clinical genomics will enable durable successful outcomes for pediatric neuro-oncology patients and this remains a dynamic area of study.

## Conclusions

The impact of NGS in the diagnosis and management of pediatric CNS tumors is still not well understood. Our single institutional experience highlights the feasibility and clinical utility of NGS in the management of pediatric neuro-oncology patients. NGS led to a change in diagnosis, the discovery of a cancer predisposition syndrome, and altered the course of treatment in a significant proportion of cases. Future prospective clinical trials using NGS are needed to establish the efficacy of molecular-based targeted therapy in children with primary and relapsed CNS tumors.
